# Neutrophil extracellular traps impair regeneration

**DOI:** 10.1111/jcmm.16896

**Published:** 2021-10-08

**Authors:** Eric Wier, Mayumi Asada, Gaofeng Wang, Martin P. Alphonse, Ang Li, Chase Hintelmann, Evan Sweren, Christine Youn, Brittany Pielstick, Roger Ortines, Chenyi Lyu, Maria Daskam, Lloyd S. Miller, Nathan K. Archer, Luis A. Garza

**Affiliations:** ^1^ Department of Dermatology Johns Hopkins University School of Medicine Baltimore Maryland USA; ^2^ Department of Plastic and Aesthetic Surgery Nanfang Hospital of Southern Medical University Guangzhou China; ^3^ Department of Molecular Biology and Genetics Johns Hopkins University Baltimore Maryland USA; ^4^ Immunology Janssen Research and Development Spring House PA USA

**Keywords:** fibrosis, Neutrophils, regeneration

## Abstract

Fibrosis is a major health burden across diseases and organs. To remedy this, we study wound‐induced hair follicle neogenesis (WIHN) as a model of non‐fibrotic healing that recapitulates embryogenesis for de novo hair follicle morphogenesis after wounding. We previously demonstrated that TLR3 promotes WIHN through binding wound‐associated dsRNA, the source of which is still unclear. Here, we find that multiple distinct contexts of high WIHN all show a strong neutrophil signature. Given the correlation between neutrophil infiltration and endogenous dsRNA release, we hypothesized that neutrophil extracellular traps (NETs) likely release nuclear spliceosomal U1 dsRNA and modulate WIHN. However, rather than enhance regeneration, we find mature neutrophils inhibit WIHN such that mice with mature neutrophil depletion exhibit higher WIHN. Similarly, Pad4 null mice, which are defective in NET production, show augmented WIHN. Finally, using single‐cell RNA sequencing, we identify a dramatic increase in mature and activated neutrophils in the wound beds of low regenerating Tlr3−/− mice. Taken together, these results demonstrate that although mature neutrophils are stimulated by a common pro‐regenerative cue, their presence and NETs hinder regeneration.

## 1 INTRODUCTION

After suffering a wound, the body initiates a well‐coordinated physiological process to restore homeostasis and re‐establish the barrier. This spontaneous process comprises four well‐studied, discrete phases: haemostasis, inflammation, proliferation and remodelling.^1^ However, the molecular details that skew repair towards fibrotic scarring, and in some cases, hypertrophic scars, rather than complete regeneration, have not been fully elucidated, despite their enormous burden on human health.^1^, ^2^, ^3^

Complete skin regeneration after wounding in mammals is rare, unlike in some animals such as urodele salamanders.^4^ It occurs via de novo hair follicle generation in mice and rabbits through a process that mimics skin embryogenesis.^5^, ^6^, ^7^, ^8^ This process of de novo follicle neogenesis (wound‐induced hair neogenesis, WIHN was first fully characterized in mice subjected to full thickness wounds.^9^ These regenerated follicles establish a distinct stem cell population, express characteristic differentiation markers, produce functional hair shafts and can complete hair cycling. In addition to hair follicles, sebaceous glands, specialized vascular and nerve supports and surrounding fat cells are regenerated.^9^, ^10^

Inflammation and different components of the immune system have been shown to promote these and other forms of regeneration in salamanders, zebrafish and even mammals, specifically injury repair and barrier function maintenance in mucosa.^11^, ^12^, ^13^ Although the cellular effects of macrophages and T cells are well studied,^14^, ^15^, ^16^, ^17^ neutrophilic effects on regeneration are not. Immediately after skin wounding, a robust inflammatory phase occurs, which allows the ingress of keratinocytes and fibroblasts to proceed afterwards. The early stage of wound healing is defined by the dramatic recruitment of mature neutrophils, which are instrumental in providing defence against microbial pathogens.^18^, ^19^, ^20^, ^21^, ^22^ This is followed by an influx of macrophages (Mφ's) that continue the phagocytic processes begun by neutrophils and aid in the transition to the proliferative phase of wound healing.^23^, ^24^, ^25^ Increasingly, macrophages have also been shown to be essential for WIHN via TNF‐induced AKT/ β‐catenin signalling.^15^, ^16^, ^17^

In addition to phagocytosis and degranulation, mature neutrophils can produce extracellular traps (ET), large extracellular web‐like structures composed of decondensed chromatin bound to various cytosolic and granule proteins.^21^, ^22^, ^26^, ^27^, ^28^ While originally recognized as a defence mechanism against pathogens,^21^, ^26^, ^27^ they have also been found to mediate ‘sterile’ inflammatory processes.^29^, ^30^ In the absence of infection, ETs can be stimulated in sterile tissue environments through various cytokines^27^, ^31^, ^32^, ^33^ and by activated platelets.^29^, ^30^ Interestingly, ETs are found within sterile wounds of mice and delay wound healing.^34^ Mechanistically, ETs are formed by the rapid decondensation of the cellular chromatin, followed by the fragmentation of the nuclear membrane and mixing of the nuclear and cytoplasmic compartments, before being expelled from the cell. The ability of neutrophils to rapidly migrate to the wound site and produce ETs, coupled with the nuclear localization of some dsRNA, made us question whether neutrophils were a source of the dsRNA critical for Toll‐like receptors 3 (TLR3)–dependent WIHN. Interestingly, while there are extensive studies on the DNA components released during ET formation,^21^, ^28^, ^35^ the RNA components are poorly understood.

Briefly, TLRs are highly conserved single‐pass membrane‐spanning receptors that recognize structurally conserved molecular components of invading microbes and activate a cascade of inflammatory signalling pathways.^36^ Rather than simply recognizing pathogen‐associated molecules, they can also initiate sterile inflammation upon recognizing damage‐associated molecular patterns (DAMPs), which are critical to recruit immune cells and initiate wound healing.^37^ TLR3 is activated by dsRNA and has primarily been studied in the context of viral infection.^38^ Mounting evidence shows that TLR3 also plays an important role in wound repair.^39^, ^40^, ^41^, ^42^, ^43^, ^44^, ^45^, ^46^, ^47^ Synthetic double‐stranded RNA (dsRNA) polyriboinosinic‐polyribocytidylic acid (poly[I:C]) treatment dramatically increases WIHN in mice. Furthermore, wound‐released dsRNA activates TLR3 to promote hair follicle regeneration.^44^ Notably, the dsRNA U1 spliceosomal small nuclear RNA (snRNA) may be an important endogenous RNA sensed via TLR3.^42^, ^43^, ^48^, ^49^ Specifically, UV damage releases U1 snRNA that stimulates cytokine production in keratinocytes and increases barrier gene transcription.^42^, ^43^

To probe how mature neutrophils influence wound regeneration and WIHN, we analysed multiple microarrays from distinct contexts of high regenerating mice and found a common neutrophil signature. Using immunofluorescence and flow cytometry, we find that neutrophils remain in the wound bed, albeit at low levels, after the acute inflammatory phase, where they produce NETs that contain the nuclear U1 dsRNA. To define how this influences regeneration, we used a neutrophil‐specific diphtheria toxin ablation model to deplete mature neutrophils in the wound bed and found that—contrary to our initial hypothesis—the absence of mature neutrophils enhances WIHN. Eliminating neutrophil's ability to produce NETs by knocking out Pad4 also boosted WIHN, confirming the negative influence of mature neutrophils on regeneration. Finally, we used single‐cell RNA sequencing to characterize WIHN‐deficient Tlr3−/− mice and found that they too have a dramatically increased population of mature neutrophils in the re‐epithelized wound bed, compared with wild‐type mice, likely contributing to their diminished regenerative capacity. These results indicate that, while important for preventing infection, mature neutrophils and their NETs negatively impact regeneration and WIHN. Although a common pro‐regenerative signal might increase neutrophil infiltration, mature neutrophils instead likely contribute to fibrosis.

## 2 MATERIALS AND METHODS

### 2.1 Mouse lines

All wild‐type and control mice used for in vivo experiments were on the C57BL/6J background. All mice were age‐matched and co‐housed until 3‐weeks of age. *Pad4* knockout mice were purchased from the Jackson Laboratory (B6. Cg‐Padi4^tm1.1Kmow^/J, 030315). The diphtheria toxin (DT)‐mediated neutrophil ablative mice were generated by crossing ROSA26iDTR (C57BL/6‐Gt(ROSA)26Sor^tm1(HBEGF)Awai^/J, 007900) and MRP8‐Cre‐ires/GFP (B6. Cg‐Tg[S100A8‐cre,‐EGFP]1Ilw/J, 021614) from the Jackson Laboratory to get heterogeneous mice and genotyped according to their specifications. Mice who genotyped positive for Cre (MRP8‐Cre+; ROSA‐iDTR^KI^) were considered PMN^DTR^ mice, while those that were negative for Cre (MRP8‐ Cre−; ROSA‐iDTR^KI^) were PMN^WT^ littermate controls.^50^ Tlr3 knockout mice (B6N.129S1‐Tlr3^tm1Flv^/J, 009675) and C57BL/6NJ controls (005304) were purchased from the Jackson Laboratory. All mice were bred and housed at an American Association for the Accreditation of Laboratory Animal Care (AAALAC)‐compliant facility, and all experimental procedures were reviewed and approved by the Johns Hopkins University Institutional Animal Care and Use Committee (IACUC).

### 2.2 Wound‐induced hair neogenesis assay

All in vivo experimental surgical procedures were performed as previously characterized.^9^, ^14^, ^44^, ^47^, ^51^ Briefly, after exposure to anaesthesia (Baxter, Isoflurane), the dorsal side of 3‐week‐old (21 days) male and female mice was shaved. Surgical scissors were used to excise 1.25 × 1.25 cm^2^ of skin on wound day 0 (WD) 0, creating wounds deep into the fascia. On approximately WD21, neogenic hair follicles in the re‐epithelialized skin tissue were quantified using reflectance confocal scanning laser microscopy (CSLM) as previously published.^44^, ^47^

### 2.3 Neutrophil depletion

Diphtheria toxin depletion was done with PMN^DTR^ and PMN^WT^ littermate control mice that were IP injected with 250 ng DT (Sigma‐Aldrich). The injections were primarily done one day before and after wounding, or on WD 6, 8 and 10.

### 2.4 Flow cytometry

Flow cytometry was used to access neutrophil depletion. Blood was collected via retro‐orbital sinus bleeds, and red blood cells were lysed RBC lysis buffer (BioLegend, 420301). Wound beds were surgically removed, and cell suspensions were prepared by digesting the tissue in a cocktail consisting of Liberase TL (Roche, 5401020001) and DNase I (Sigma, DN25) in RPMI 1640 (Gibco, 11875093). Cells were washed and then Fc blocked (BioLegend, 101320), before staining with an antibody cocktail (Table 1). Finally, cells were washed and resuspended in FACS buffer containing propidium iodide (Miltenyi, 130‐093‐233). All flow cytometry experiments were performed on a BD LSR II, and downstream analysis of data was performed using FlowJo.

**TABLE 1:** Flow cytometry antibodies

Name	Host	Fluorophore	Manufacturer/Product #
MHCII (IA/IE)	Rat	BV421/Pacific Blue	BioLegend/107631
CD3	Rat	BV510/AmCyan	BD/740147
Ly6C	Rat	FITC	BD/553104
Ly6G	Rat	PE	BioLegend/127607
CD45	Rat	PE‐Cy5.5	Invitrogen/35‐0451‐82
CD115	Rat	PE‐Vio770	BioLegend/135523
CD11c	Hamster	APC	BioLegend/117310
CD11b	Rat	APC‐Cy7	BD/557657

### 2.5 Neutrophil extracellular trap measurement

Wound beds were surgically removed at WD2 and WD7, using a 6‐mm biopsy punch to remove excess tissue. Cell suspensions were prepared by digesting the tissue in a cocktail consisting of Liberase TL (Roche, 5401020001) and DNase I (Sigma, DN25) in RPMI 1640 (Gibco, 11875093). Cells were washed and then Fc blocked (BioLegend, 101320), before staining with an antibody cocktail containing anti‐MPO (Abcam, ab208670, 1:500) or respective isotype control (Abcam, ab172730, 1:500). Cells were then stained with a secondary Alexa Fluor 647 antibody (Abcam, ab150083, 1:2000). On the final wash, SYTOX green was added (Thermo Fisher, S7020, 1:1000). This was performed on a BD LSR II, and downstream analysis of data was performed using FlowJo.

### 2.6 3′‐end single‐cell RNA sequencing

The re‐epithelialized wound beds (WD10) of a Tlr3−/− and a C57BL/6NJ control mouse were excised, and cell suspensions were prepared by digesting the mouse skin tissue in a cocktail consisting of Liberase TL (Roche, 5401020001) and DNase I (Sigma, DN25) in RPMI 1640 (Gibco, 11875093). Propidium iodide and DAPI‐positive dead cells were removed via cell sorting with a BD FACSAria II. Single‐cell libraries were prepared via a 10× Genomics Chromium Single‐Cell Platform, followed by sequencing using Illumina NovaSeq 6000. The results were run through Cell Ranger pipeline software for sequence alignment and basic filtering. GEM generation, barcoding, cDNA amplification, library preparation, quality control and sequencing were performed at the Genomics High Throughput Sequencing facility at Johns Hopkins School of Medicine.

Downstream analysis, after the Cell Ranger pipeline, was done using the Seurat R package. A standard pre‐processing workflow was done, removing low‐quality cells or doublets, filtering unique feature count over 3750 and below 200, as well as filtering out cells with higher than 5% mitochondrial counts. This resulted in 4150 WT and 5648 Tlr3−/− cells for downstream bioinformatics. Expression matrices then underwent normalization, scaling, principal components analysis and subsequent t‐SNE analysis using Seurat packages. Seurat was then used to generate conserved genes, differentially expressed genes, feature plots, dot plots and ridge plots. Cell clusters were then defined querying conserved genes and differentially expressed genes against the ImmGen gene expression database (www.immgen.org) using the interactive tool ‘My Gene Set’.

### 2.7 Histology

Biopsies from mouse skin tissue were removed and fixed in 4% paraformaldehyde overnight and then transferred to 70% ethanol. Samples were then submitted to the Johns Hopkins Oncology Tissue Services Core facility where they were embedded in paraffin. Tissue sections were obtained at 4 μm thickness and mounted onto glass slides, followed by haematoxylin and eosin (H&E) staining.

### 2.8 Immunofluorescence and immunohistochemistry

Immunofluorescence (IF) microscopy was performed on de‐paraffinized tissue sections that received heat‐induced antigen retrieval using Target Retrieval Solution (Agilent Dako, S169984‐2). After washing and permeabilization in TBS‐T universal buffer (0.2% Triton X‐100 in Tris‐buffered saline), sections were blocked at room temperature in 5% goat, donkey or foetal bovine serum with 1% bovine serum albumin. Tissue sections were then incubated overnight at 4°C with primary antibodies (Table 2) in Antibody Diluent (Agilent Dako; S080983‐2). Following a wash step, sections were incubated in fluorescent‐dye‐conjugated secondary antibodies diluted in antibody diluent for 1 h at room temperature. After final washing, sections were mounted with VECTASHIELD^®^ Hardset™ Antifade Mounting Medium with DAPI (Vector Laboratories, H‐1500) for nuclear staining. All imaging was done on a DFC365FX (Leica) at 20× and 40× magnifications.

**TABLE 2:** Immunofluorescence and immunohistochemistry antibodies

Name	Host	Dilution	Company/ Product #
MPO	Goat	1:200	Abcam/ab208670
F4/80	Rat	1:200	Abcam/ab6640
Ly6G	Rat	1:200	BioXCell/BP0075‐1
H3Cit	Rabbit	1:500	Abcam/ab5103
Alexa Fluor^®^ 488 Anti‐Goat IgG (H + L)	Rabbit	1:1000	Invitrogen/A27012
Alexa Fluor^®^ 488 Anti‐Rabbit Ig (H + L)	Goat	1:1000	Invitrogen/A‐11008
Alexa Fluor^®^ 594 Anti‐Rabbit IgG (H + L)	Goat	1:1000	Invitrogen/A‐11037
Alexa Fluor^®^ 488 Anti‐Rat Ig (H + L)	Goat	1:1000	Invitrogen/A‐11006
Alexa Fluor^®^ 594 Anti‐Rat Ig (H + L)	Donkey	1:1000	Invitrogen/A‐21209

### 2.9 U1 in situ hybridization

U1 in situ probes were designed and ordered in the Stellaris Probe Designer (Biosearch Technologies) (Table 3). Tissue sections were de‐paraffinized and stained following Biosearch Technologies Stellaris RNA FISH protocol for formalin‐fixed paraffin‐embedded tissue. Briefly, tissue sections were washed in Wash Buffer A (Biosearch Technologies, SMF‐WA1‐60), before adding 200 μl hybridization buffer (Biosearch Technologies, SMF‐HB1‐10) containing the U1 probe and covering the tissue with a glass coverslip. The slides were then incubated overnight in a humid box at 37°C. Slides were then immersed in Wash Buffer A in the dark at 37°C for 30 min, allowing the coverslips to float off. Slides were then washed for 5 min with Wash Buffer B (Biosearch Technologies, SMF‐WB1‐20), before sections were mounted with VECTASHIELD^®^ Hardset™ Antifade Mounting Medium with DAPI (Vector Laboratories, H‐1500) for nuclear staining. All imaging was done on a DFC365FX (Leica) at 63× magnifications.

**TABLE 3:** U1 in situ hybridization probes

Sequence Name	Sequence
U1 snRNA_1	cccctgccaggtaagtat
U1 snRNA_2	caccttcgtgatcatggt
U1 snRNA_3	aagcctcgccctgggaaa
U1 snRNA_4	acatccggagtgcaatgg
U1 snRNA_5	gggaaatcgcaggggtca
U1 snRNA_6	cagtcgagtttcccacat
U1 snRNA_7	ccccactaccacaaatta
U1 snRNA_8	aggggaaagcgcgaacgc

### 2.10 Microarray, RNA‐seq and proteomic analysis

For both Rnasel−/− and WT mice, total RNA was isolated from mouse tissue at scab detachment (SD) day 0 (also WD10) from the wound. RNA was submitted to the JHMI Deep Sequencing & Microarray core facility and profiled using the Affymetrix Clariom™ S mouse array platform, according to the manufacturer's protocols. Gene chips were scanned, generating CEL pixel intensity files, which were processed and analysed using Partek^®^ Genomics Suite™ software, and the Robust Multichip Analysis (RMA) algorithm was used for normalization. RNaseL null mouse microarrays are available in GSE164003 NCBI GEO. GF and SPF microarrays are available in GSE158613 NCBI GEO. For SPF and GF analysis, total RNA from early wound bed skin (~WD12) was submitted to the JHMI Transcriptomics and Deep Sequencing Core. The 1.0ST exon sequencing of mouse RNA was performed according to the manufacturer's standard protocol. The raw affymetrix CEL data were standardized using Robust Multichip Analysis (RMA) algorithm for comparison.

Proteins from the wound centre and wound edges were analysed by proteomics, as previously described.^47^ Briefly, after saline washing, samples were lysed in 5% sodium deoxycholate (DOC) detergent. After sequential peptide processing (reduction, alkylation and trypsinolysation), downstream resolving and analysis were performed on a nanoACQUITY UPLC system with a Tribrid Orbitrap‐quadrupole‐linear ion trap mass spectrometer (Thermo Fisher). The UniProt mouse reference proteome was used to align tandem mass spectra (MS‐MS) data in conjunction with the SEQUEST HT algorithm. Protein abundance ratios were calculated by comparing MS1 peptide ion intensity peaks. Machine‐learning‐based software (Percolator) was used for peptide identification and validated at an FDR of at least 0.05. Centre vs. edge proteomics is available at the data repository of University of Maryland Metallotherapeutics Research Center, Baltimore (https://bit.ly/2U9qwqO), and the mass spectrometry proteomic data have been deposited to the ProteomeXchange Consortium via the PRIDE partner repository with the data set identifier PXD013854.

### 2.11 Quantification and Statistical analysis

All in vivo and in vitro experiments were performed in at least individual instances. Univariate statistical analysis was performed using Student's *t* test, and multivariate analysis was performed using ANOVA. All statistical analyses and graphical representations were generated using GraphPad Prism software. Statistical significance is defined as *p*‐values <.05 derived from the standard error of mean calculations.

## 3 RESULTS

### 3.1 Neutrophil signature present during skin regeneration

**FIGURE 1:**
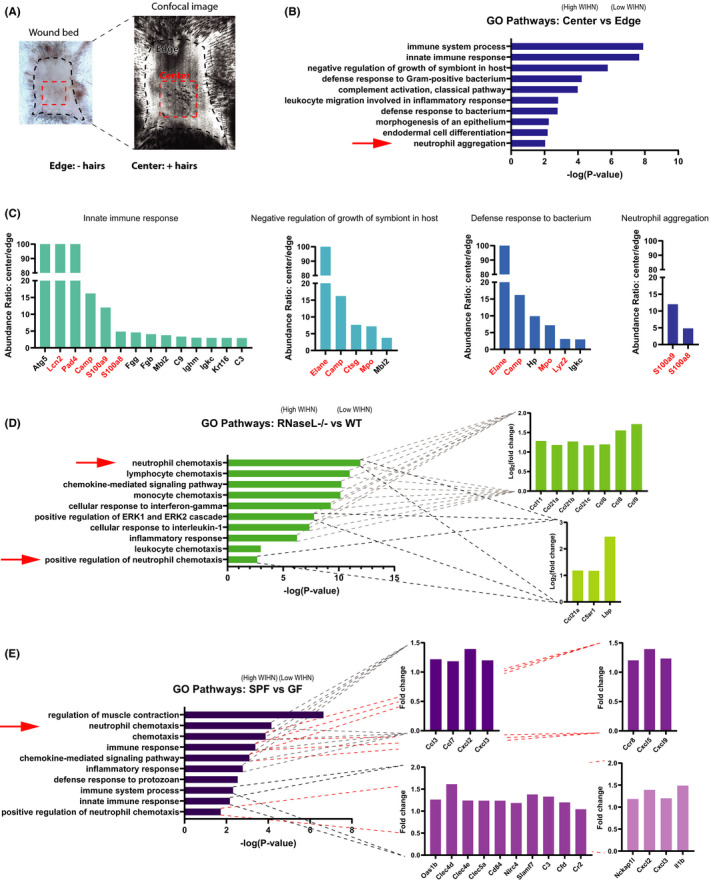
Neutrophil signatures correlate with high Wound‐induced Hair Neogenesis (WIHN) in multiple models. (A) Schematic of hair neogenesis preferential localization to wound centre (high WIHN) rather than wound edge (low WIHN); image duplicated from Figure 3F for clarity. (B) Proteomic gene ontology (GO) analysis of the top 100 genes wound centre vs. wound edge in wild‐type (WT) mice shows a predominance of innate immune response pathways and neutrophil signatures in the wound centre. (C) Abundance ratios of genes from select GO terms highlighted in b. show enrichment in the wound centre of antimicrobial and granular proteins, labelled in red. (D) GO analysis of the top 200 genes from high WIHN Rnasel^−/−^ vs. low WIHN WT mice shows a predominance of neutrophil and innate immune cell chemotaxis pathways in Rnasel^−/−^ mice. Inset graphs show the gene fold expression changes for genes present in that category. (E) GO enrichment analysis of the top vs. bottom 500 differentially expressed genes between specific pathogen‐free (SPF; high WIHN) and germ‐free (GF; low WIHN) mice demonstrates higher neutrophil chemotaxis and innate immune categories in SPF mice (*n* = 3 independent animals per group). Inset graphs show the gene fold expression changes for genes present in that category

To begin to characterize the role of neutrophils in Wound‐induced hair neogenesis (WIHN), we performed bioinformatic analysis on previous microarrays of multiple high regenerating mouse models, probing for innate immune and neutrophil signatures. First, we analysed the proteome comparing the centre of the re‐epithelialized wound bed (high WIHN) to the surrounding periphery/edge (low WIHN) (Figure 1A).^47^ Gene ontology analysis revealed that, in the area of high WIHN, neutrophil aggregation and other defence pathways against bacterium are enriched, characterized by an abundance of antimicrobial and granular proteins, such as neutrophil elastase (Elane), cathelicidin (Camp), and myeloperoxidase (Mpo), all known to be elevated in neutrophils (Figure 1B,C). In a second model system, high WIHN Rnasel−/− mice (manuscript in review), at time ~WD11 (day of wound closure), are also enriched in genes associated with neutrophils. Neutrophil chemotaxis is the most significant upregulated gene ontology category, with other chemotactic and inflammatory pathways up, as well (Figure 1D). Finally, we analysed the gene expression changes between wounded specific pathogen‐free (SPF) mice, which have increased regeneration and WIHN, when compared to germ‐free (GF) mice at WD11.^52^ Like the other two high regeneration models, when compared to GF, SPF mice have elevated neutrophil chemotaxis and immune response transcripts (Figure 1E). Together, these unique examples correlate neutrophil chemotaxis with high regeneration and WIHN.

### 3.2 Neutrophils persist in the wound bed after the acute inflammatory phase, producing extracellular traps

**FIGURE 2:**
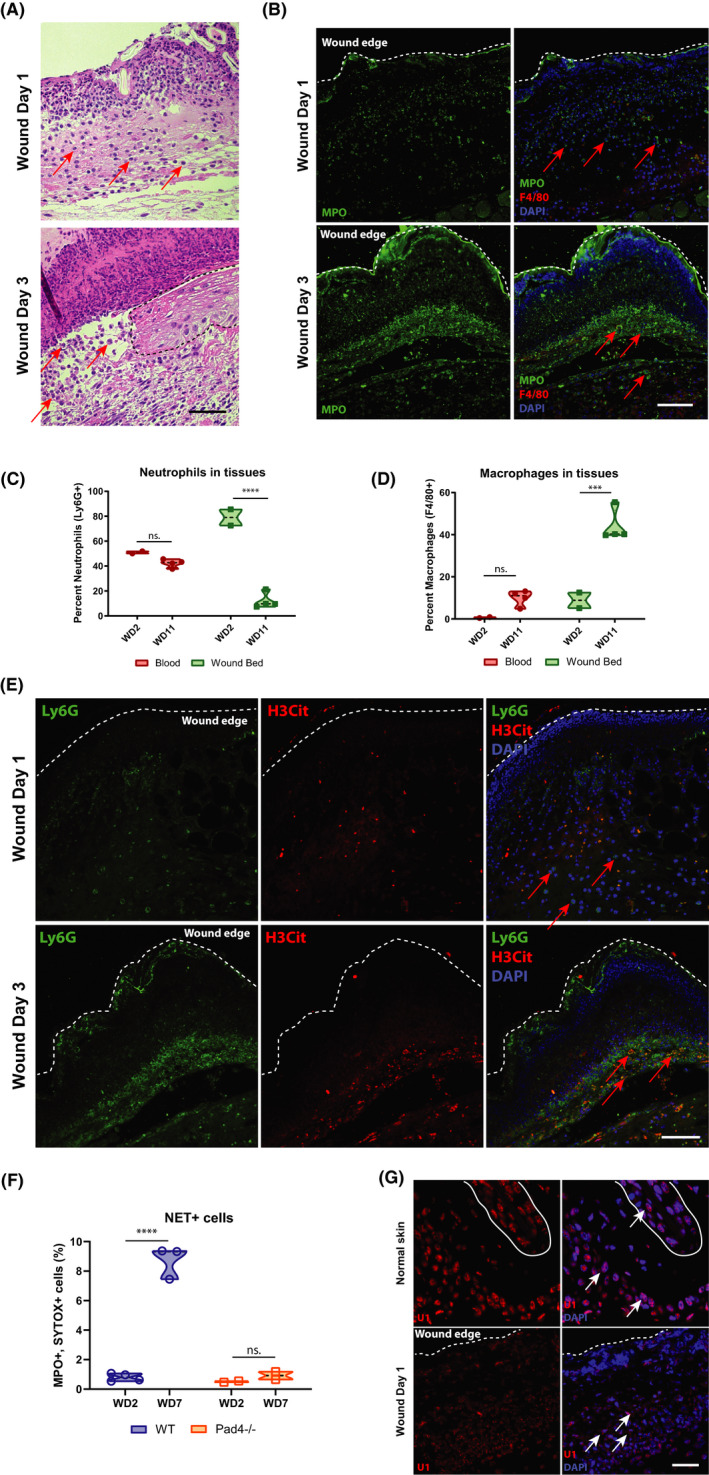
Neutrophils persist in wound bed after the acute inflammatory phase, producing extracellular traps. (A) Neutrophils are present in the wound beds of C57BL/6J mice at early time points, as visible in representative haematoxylin and eosin (H&E) staining. Arrows indicate regions of interest, and dashed line demarcates boundaries. Black scale bar = 50 µm. (B) Neutrophils predominate throughout the wound beds of C57BL/6J mice on wound day (WD) 1 and WD3, visible in prominent myeloperoxidase (MPO) immunofluorescence (IF) staining (green). Few macrophages are present (Red, F4/80). White scale bar = 200 µm. (C) Per cent neutrophil (Ly6G+ cells from total CD45+ cells) levels are consistent in the blood throughout the wound time course but drop in the wound bed at WD11, as measured by FACS. ****p < 0.0001, as calculated by two‐way ANOVA. *N* = 2 vs. 4. Results are representative of at least two independent experiments. (D) Macrophage (F4/80) levels are largely absent from the blood and low in the wound bed during the early phase of healing but increase dramatically at WD11, as measured by FACS. ***p < 0.004, as calculated by two‐way ANOVA. n.s., not significant. *N* = 2 vs. 4. Results are representative of at least two independent experiments. (E) Citrullinated histone H3 (H3Cit, red) co‐localized with Ly6G+ neutrophils (green), beginning at WD3 in the wound beds of IF‐stained C57BL/6J mice, indicating the formation of extracellular traps. (F) Neutrophil extracellular trap‐positive cells (MPO+, SYTOX green +) are present at late wound time points, but are absent in the wound beds of PAD4−/− mice, as measured by FACs. ****p < 0.0001, as calculated by two‐way ANOVA. *N* = 7 vs. 4. Results are representative of at least two independent experiments. (G) Cytoplasmic U1 snRNA is present in the wound bed of C57BL/6J mice, while it localized exclusively in the nuclei of unwounded controls, as visualized by representative FISH. The solid white line delineates a hair follicle. White scale bar = 80 µm

Having correlated the presence of neutrophils with high WIHN in 3 disparate contexts (Figure 1), we next characterized mature neutrophil infiltration in the wound beds of C57BL/6J mice after wounding. As anticipated, neutrophils are abundant in the acute phase, wound days (WD) 1–3 of the healing process as seen by haematoxylin and eosin (H&E) staining (Figure 2A) and immunofluorescence (IF) of myeloperoxidase (MPO), a major neutrophil granule protein (Figure 2B). While neutrophils predominate at early time points, they persist in the wound bed as late as WD11, the common day when re‐epithelization is complete and the scab has detached (Figure 2C). This persistence of neutrophils beyond the classic inflammatory phase of wound healing is consistent with the late time points measured in Figure 1 demonstrating a neutrophil signature. In contrast to the early infiltration of neutrophils, macrophages accumulate starting at WD3 (Figure 2B) but remain a major component of the wound even after re‐epithelization (Figure 2D). These results show the dynamic changes in immune cell infiltration, but with an underappreciated persistence of some neutrophils late in wound healing, notably at the time of morphogenesis.

Given the importance of dsRNA to promote WIHN and the above association of neutrophils with high WIHN, we hypothesized that neutrophil extracellular traps (NETs) release of neutrophil nuclear content might release both dsDNA and dsRNA from the nucleus to promote WIHN. We visualized citrullinated histone H3 (H3Cit) to identify NETs as early as WD3 (Figure 2E). The production of NETs is mediated by Pad4, an enzyme that modifies arginine residues on histones to citrulline, changes their charge and triggers massive chromatin decondensation.^53^, ^54^ Consistent with this, NETs are virtually absent in mice lacking Pad4, while abundant in the wound bed after the acute phase of healing in WT mice (Figure 2F). Although NETs are characterized by their extruded DNA, which form web‐like scaffolds containing cytosolic and granular proteins, little is known about NET's RNA content and associated effects on WIHN. Given that U1 small nuclear (sn) RNA is proposed as a TLR3 agonist damage‐associated molecular pattern (DAMP) important for skin barrier repair,^42^, ^43^ we visualized it by fluorescence in situ hybridization (FISH). U1 snRNA is present throughout unwounded tissue of C57BL/6J mice and is located nuclearly (Figure 2G), whereas, in wounded mice, U1 snRNA translocates from lobulated neutrophil nuclei to the cytoplasm, suggesting its progressive release. Consistent with this, we also note a fine haze of signal extracellularly. Taken together, these data suggest a model where neutrophils, extracellular traps and U1 dsRNA persist to the morphogenesis stage of wound healing and might modulate WIHN.

### 3.3 Mature neutrophils inhibit wound‐induced hair neogenesis

**FIGURE 3:**
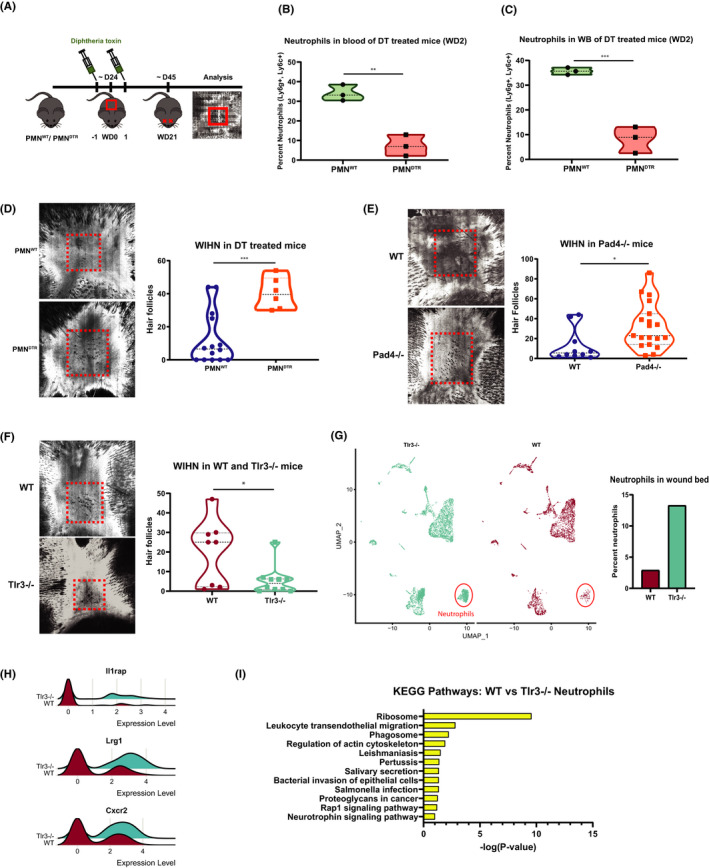
Mature neutrophils inhibit WIHN. (A) Schematic of neutrophil depletion via diphtheria toxin (DT, 250 ng) injection in heterozygous ROSA26iDTR/MRP8‐Cre‐ires mice (PMN^DTR^ and PMN^WT^). (B) Diphtheria toxin injection in heterozygous ROSA26iDTR/MRP8‐Cre‐ires mice (PMN^DTR^ and PMN^WT^), following the injection scheme in (A), successfully depletes neutrophils from the blood in mice with large wounds. Fold = −4.33. **p = 0.0098. *N* = 3 vs. 3. Results are representative of at least two independent experiments. (C) Mice treated as in (A) are depleted of neutrophils in their wound beds at WD2. Fold = −4.27. **p = 0.0069. *N* = 3 vs. 3. Results are representative of at least two independent experiments. (D) PMN^DTR^ mice IP injected with diphtheria toxin (DT, 250 ng) on WD‐1 and WD1 exhibit increased WIHN (CSLM, images; fold = 3.23, ***p = 0.0010, *N* = 14 vs. 6). In each image, the dash red box indicates the area of hair follicle regeneration. (E) Pad4^−/−^ mice defective in extracellular traps exhibit increased WIHN (CSLM, images; fold = 2.47, *p* = 0.026, *N* = 10 vs. 19 (F) Tlr3^−/−^ mice exhibit decreased WIHN (fold = −3.75 *p* = 0.0218, *N* = 8 vs. 10). (G) The presence of increased mature neutrophils correlates with decreased WIHN in TLR3^−/−^ mice. scRNA‐seq t‐SNE plot shows differences between Tlr3−/− (blue, 4843 cells) and WT (red, 3172 cells) wound beds at WD 10. The plots were generated via Seurat. The neutrophil cluster is circled in red. Per cent of neutrophils is significantly different in Tlr3−/− vs. WT mice as graphed to bottom right. ****p < 0.0001, as calculated by chi‐square test. Neutrophils have the greatest fold change (fold = 6.98) between Tlr3−/− and WT mice, graphed to bottom left. (H) Neutrophil‐associated gene expression is more pronounced within the neutrophils of Tlr3−/− mice, compared to WT. Generated in Seurat with RidgePlot function. (I) Kyoto Encyclopedia of Genes and Genomes (KEGG) pathway gene ontology analysis of the top 50 differentially of the mature neutrophil cluster demonstrates categories associated with neutrophil activation preferentially in Tlr3 −/− mice compared with WT

To functionally test the importance of neutrophils, we generated a transgenic mouse model for selective and inducible ablation of neutrophils upon injection of diphtheria toxin (DT).^50^ MRP8‐Cre mice expressing Cre recombinase under the control of the neutrophil‐associated human MRP8 promoter^55^, ^56^, ^57^ were crossed with ROSA‐iDTR^KI^ mice, which have a Cre‐inducible simian DT receptor (DTR),^58^ yielding progeny that suffer selective cell death in DTR expressing mature neutrophils with DT treatment. Neutrophil ablated mice in this model have intact monocyte and macrophage populations (Figure S1).^50^ After injections—one before wounding and one a day after—we saw substantially reduced neutrophil numbers in the blood (fold = −4.33) and wound beds (fold = −4.27) of PMN^DTR^ (MRP8‐Cre+, ROSA‐iDTR^KI^) mice at WD2 and no differences in control mice (PMN^WT^ mice: MRP8‐Cre−, ROSA‐iDTR^KI^) (Figure 3B,C). This decrease in wound bed neutrophils occurs even with the standard large size used to test for WIHN (Figure 3C). In line with the above findings, the reduction in mature neutrophils correlates with substantially elevated WIHN (fold = 3.23) (Figure 3D). Later neutrophil ablation in the healing process yielded similar results (WD6, 8 and 10) (Figure S2). Collectively, these data suggest that mature neutrophils have a detrimental effect on the regeneration of hair follicles.

Finally, given the presence of NETs late in wound healing (Figure 2F), we sought to test the role of NETs in hair follicle neogenesis directly. We, therefore, tested WIHN in NET‐deficient Pad4−/−  mice, as employed in Figure 2F. In the absence of Pad4 and NETs, WIHN is enhanced (fold = 2.47, *p* = 0.026) (Figure 3E). This suggests that NETs reduce the regenerative capacity of mice during the wound‐healing process.

### 3.4 Single‐cell RNA‐seq correlation of mature neutrophils with poor WIHN

We next wondered whether increased neutrophils and NETs might similarly play inhibitory roles in other contexts of poor regeneration. Double‐stranded RNA sensing, mediated by Tlr3 and downstream effector pathways Il‐6/ Stat3, has been shown to be critical for WIHN. Tlr3−/− mice, though grossly normal without wounding, have substantially less regenerated hair follicles than their wild‐type controls.^44^ Although TLR3 dsRNA sensing has been shown to be critical for neutrophil recruitment and NET production in a model for acute lung injury (ALI) and glomerulonephritis (GN), we probed to see whether Tlr3−/− mice paradoxically have increased neutrophil levels, contributing to lower WIHN.^59^, ^60^ Consistent with our previous published data, Tlr3−/− mice have substantially less WIHN than their WT controls (fold = −3.75, *p* = 0.0218) (Figure 3F). Given successful published literature on normal wild‐type single‐cell RNA‐seq changes during wounding,^61^ we then performed single‐cell RNA sequencing on wild‐type and Tlr3−/− re‐epithelialized wound beds at WD10. Approximately 8015 sequenced cells met standard quality control metrics and were further analysed in the Seurat R package.^62^ Unsupervised clustering and UMAP non‐linear dimensional reduction identified 18 cell clusters. Seurat generated conserved and differentially expressed genes, which were used to assign cluster identities (Figure S3a). Keratinocytes and fibroblasts did have differentially expressed genes (Table S1). As anticipated from our earlier data where inhibiting mature neutrophils promotes WIHN, we find the mature neutrophil cluster in the low regenerating Tlr3−/− mice to be significantly different (*p* < 0.0001) and most substantially increased (fold = 6.98) relative to WT mice (Figure 3G, Figure S3b). There are also marked changes in the fibroblast (Fibro1) cluster (fold = 5.12), which is also significantly different (*p* < 0.0001) from WT mice (Figure 3G, Figure S3c). Consistent with increased neutrophils in low regenerating TLR3 −/− mice, neutrophil‐associated genes are significantly elevated in Tlr3 −/− mice (Figure 3H), with a general elevation in gene ontology categories consistent with greater neutrophil activation in Tlr3 −/− such as ribosome‐associated transcripts (Figure 3I). Together, these data demonstrate that elevated mature and activated neutrophil levels in Tlr3−/− mice correlate with their low WIHN.

## 4 DISCUSSION

The wound‐healing process is a careful balance of interconnected steps that weigh the benefits of quick barrier repair, which leads to fibrous scarring, and more complete regeneration, that restores function and appearance. While the role inflammation plays in regeneration and scarring is still being elucidated, increasing evidence suggests that neither excess nor lack of inflammation supports regeneration. Fgf9‐producing γ‐δ T cells are critical for WIHN, infiltrating into wound bed immediately before re‐epithelialization and onset of hair follicle regeneration.^14^ Macrophages have also been shown to be important in the process, with their ablation eliminating WIHN.^15^, ^16^, ^17^ Additionally, the injection of the dsRNA mimic poly(I:C), as early as WD3, dramatically enhances WIHN.^44^ Intriguingly, spiny mice (*Acomys*) have a dramatically reduced inflammatory response postwounding, with less cytokines and virtually no macrophages until late in the process, when compared to laboratory mice (*Mus*), despite having substantially improved regeneration.^63^, ^64^ Consistent with this idea of a complex network of inflammatory cells influencing regeneration, we show that mature neutrophils persist in the wound bed after the acute inflammatory phase and—despite multiple correlations between high WIHN and neutrophils—have a detrimental effect on regeneration in our functional model.

Using histological and flow cytometry techniques, we show that while abundant immediately after wounding, mature neutrophils remain in low levels within the wound bed after re‐epithelization. These late‐stage neutrophils produce NETs, which are lacking in mice that are deficient in Pad4, an enzyme critical for chromatin decondensation and NET formation.^53^, ^54^ Significantly, Pad4−/− mice have increased WIHN, when compared with WT mice, which correlates with the capacity of NETs to damage tissue in diseases such as small vessel vasculitis,^65^ systemic lupus erythematosus,^66^, ^67^, ^68^, ^69^, ^70^ rheumatoid arthritis^71^ and psoriasis.^72^ Furthermore, selective genetic neutrophil ablation dramatically boost WIHN. Mice deficient in dsRNA sensing Tlr3 have severely reduced WIHN and substantially more mature neutrophils present in the re‐epithelized wound bed at the time point immediately preceding regeneration. All these data suggest a model where neutrophils play an important role in defence against bacterial pathogens but whose persistence within the wound bed too long after barrier repair hinders regeneration. In this sense, the dramatic decrease in neutrophil numbers in the course of wound healing is likely important for later WIHN. In the future, it will be interesting to see whether selectively targeting NETs (eg, PAD4 inhibitors, DNase I, N‐acetylcysteine) enhance regenerative wound repair.

Our work suggests important areas of future investigation. One question is unravelling the paradox of why neutrophil infiltration signatures correlate with high WIHN, but mature neutrophils inhibit WIHN. One possible model is that a common upstream cue or factor both promotes WIHN and promotes neutrophil infiltration, but the latter serves to limit WIHN in favour of decreasing infection risk. Defining this common upstream signal will be important for future work.

It will also be interesting to define the function of neutrophils within the re‐epithelized wound since the barrier has been restored. It is possible that the neutrophils present at later time points in the healing process are not the classical pro‐inflammatory N1 subtype of neutrophils, but more recently discovered anti‐inflammatory N2 neutrophils. Initially identified in cancer, N2 neutrophils have impaired anti‐tumour capacity and express gene characteristic of alternatively activated M2 macrophages, such as arginase‐1 (Arg1) and mannose receptor C‐type 1 (Mrc1).^73^ Studies in myocardial infarction show that neutrophils are temporally polarized between the two subtypes, beginning as N1 neutrophils and shifting towards N2 as inflammation recedes and tissue repair proceeds.^74^ Additionally, recent evidence shows that a related subtype of immature neutrophil can promote axon regeneration post‐crush injury to the optic nerve of mice.^75^ For this reason, it will be important to continue to classify the subtypes of neutrophils present throughout WIHN, rather than focusing sole on mature inflammatory neutrophils.

Another question is whether nuclear RNAs released in NETs have any function besides the general theorized one for released DNA. Since NETs inhibit WIHN, nuclear dsRNA release in NETs might act differently from exogenous or other endogenous dsRNA; perhaps dsRNA in NETs is trapped and not released effectively to stimulate a dsRNA response.

In summary, we demonstrate a novel role for NETs and mature neutrophils to inhibit regeneration (Figure S4). Future studies will be important to further understand the biology of regeneration and test the capacity for neutrophil inhibition to promote regenerative healing.

## CONFLICT OF INTEREST

None of the authors have any conflict of interest regarding this manuscript.

## AUTHOR CONTRIBUTIONS

**Eric Wier:** Conceptualization (lead); Data curation (lead); Formal analysis (lead); Methodology (lead). **Mayumi Asada:** Investigation (supporting). **Gaofeng Wang:** Investigation (supporting). **Martin Alphonse:** Investigation (supporting). **Ang Li:** Investigation (supporting). **Chase Hintelmann:** Investigation (supporting). **Evan Sweren:** Investigation (supporting). **Christine Youn:** Investigation (supporting). **Brittany Pielstick:** Investigation (supporting). **Roger Ortines:** Investigation (supporting). **Chenyi Lyu:** Investigation (supporting). **Maria Daksam:** Investigation (supporting). **Lloyd Miller:** Investigation (supporting). **Nathan K. Archer:** Investigation (supporting). **luis garza:** Conceptualization (lead); Formal analysis (lead); Funding acquisition (lead); Project administration (lead); Supervision (lead); Writing‐review & editing (lead).

## COMPETING FINANCIAL INTERESTS

L.S.M. is a full‐time employee of Janssen Pharmaceuticals and may hold Johnson & Johnson stock and stock options. L.S.M. performed all work at his prior affiliation at Johns Hopkins University School of Medicine, and he has received prior grant support from AstraZeneca, Pfizer, Boehringer Ingelheim, Regeneron Pharmaceuticals and Moderna Therapeutics, was a paid consultant for Almirall and Janssen Research and Development, was on the scientific advisory board of Integrated Biotherapeutics and is a shareholder of Noveome Biotherapeutics, which are all developing therapeutics against infections and/or inflammatory conditions.

## Supporting information

Supplementary Material

## Data Availability

All gene expression data sets have been uploaded to NCBI GEO and are available at the above accession numbers listed in the Methods section.
