# Supplementary material for: Neutrophil extracellular traps impair regeneration
Source: J Cell Mol Med. 2021 Oct 8;25(21):10008–19. doi: 10.1111/jcmm.16896 (PMC8572775; doi:10.1111/jcmm.16896)
Supplement: Supplementary file 1 — Supplementary Material [file JCMM-25-10008-s001.pdf]

S1

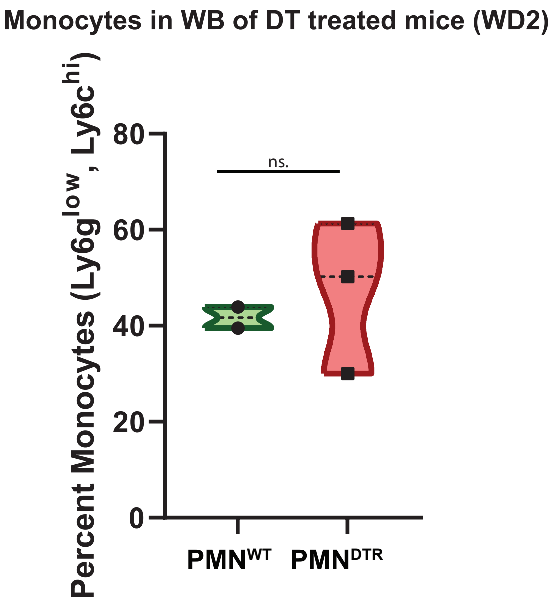

S1. PMN^{DTR} mice IP injected with diphtheria toxin (DT, 250ng) on wound days -1 and 1 have normal levels of monocytes within the wound bed. ns. = not significant. N = 2 vs. 3.

S2

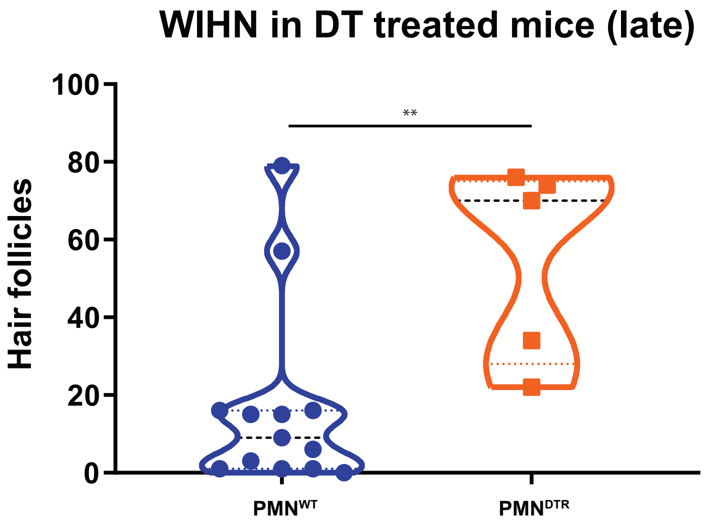

S2. PMN^{DTR} mice IP injected with diphtheria toxin (DT, 250ng) on wound days 6, 8, and 10 exhibit increased WIHN (CSLM, images; fold = 3.28, **p = 0.0085, N = 13 vs 5).

A.

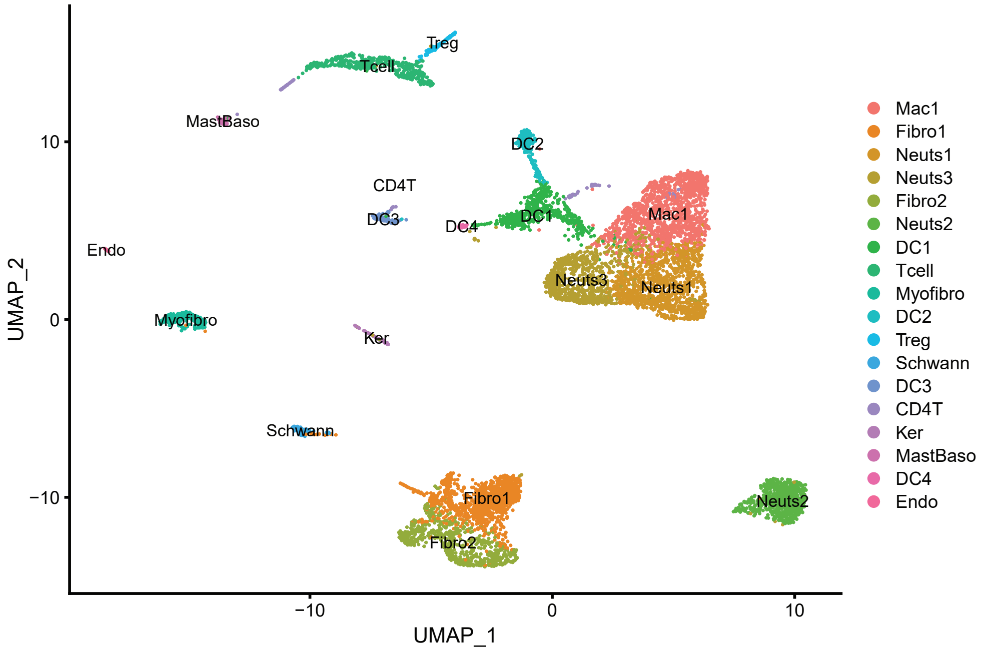

B.

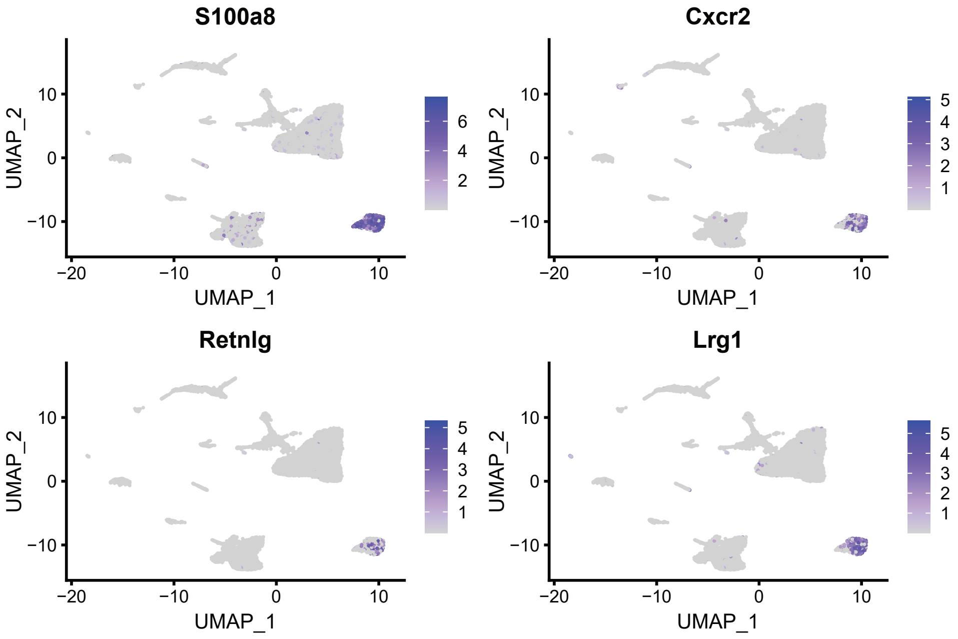

C.

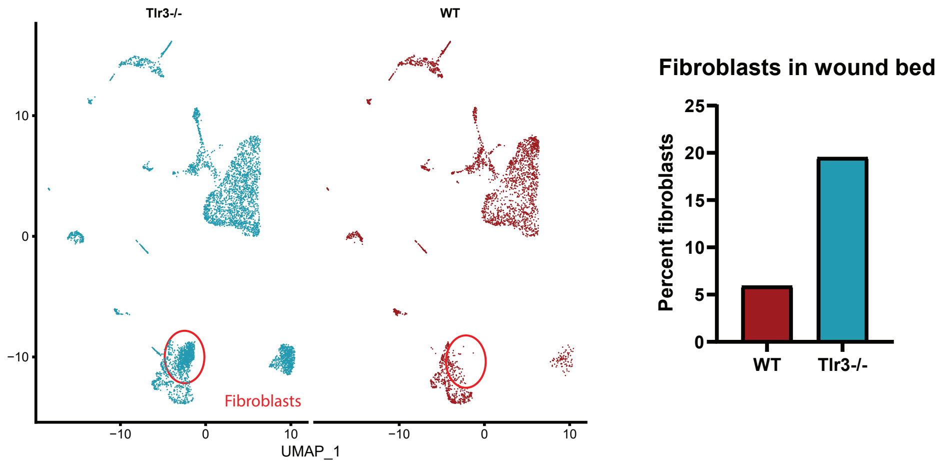

S3: a. Neutrophil associated genes used to identify the neutrophil cluster in UMAP non-linear dimensional reduction, via Seurat R package, of WT and Tlr3-/- mice. b. UMAP plot showing cellular heterogeneity with 18 distinict cell clusters identified and color coded.

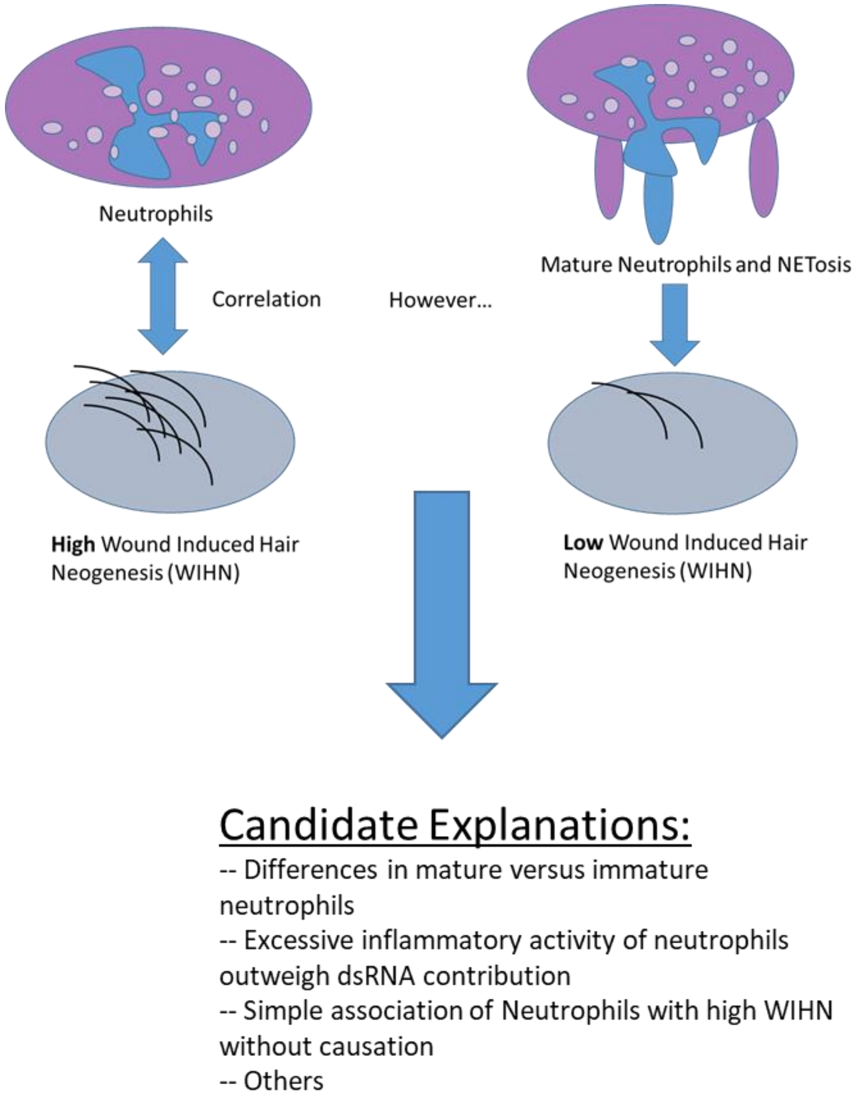

S4: Graphical Abstract for Manuscript

Supplementary Table 1: Differentially expressed genes in TLR3KO scRNAseq Wounding

Fibro1 diff expressed genes (TLR3KO/wt)

		p_val	avg_logFC	pct.1	pct.2	p_val_adj
1	Gm42418	3.52E-53	-0.91236717	0.92	0.999	1.09E-48
2	Cd74	4.89E-17	-1.062660212	0.171	0.52	1.52E-12
3	Thbs1	3.49E-16	-0.818274581	0.262	0.61	1.08E-11
4	Lyz2	2.19E-15	-0.929151347	0.182	0.502	6.80E-11
5	Lars2	9.68E-14	-0.771531228	0.299	0.582	3.01E-09
6	Apoe	1.50E-13	-0.760849969	0.193	0.461	4.67E-09
7	Il1b	1.48E-12	-1.315098057	0.059	0.306	4.58E-08
8	Ubb	2.15E-11	0.551482442	0.727	0.546	6.68E-07
9	Rps29	4.51E-11	-0.337758885	0.856	0.929	1.40E-06
10	AY036118	7.08E-11	-0.561067335	0.337	0.619	2.20E-06
11	Hsp90ab1	1.13E-10	0.412788175	0.813	0.702	3.50E-06
12	B2m	2.05E-10	-0.733449473	0.267	0.511	6.36E-06
13	S100a9	2.41E-10	-1.387746037	0.011	0.204	7.50E-06
14	H2-Ab1	4.09E-10	-0.905331404	0.096	0.327	1.27E-05
15	H2-Aa	4.87E-10	-0.940155584	0.102	0.328	1.51E-05
16	H19	8.03E-10	-0.428795005	0.465	0.737	2.49E-05
17	Postn	8.60E-10	-0.282747842	0.85	0.977	2.67E-05
18	Ctss	1.03E-09	-0.879803927	0.059	0.268	3.21E-05
19	H2-Eb1	3.01E-09	-0.928069509	0.091	0.299	9.36E-05
20	Psap	9.31E-09	-0.608773971	0.31	0.519	0.000289

Keratinocytes diff expressed genes (TLR3KO/wt)

		p_val	avg_logFC	pct.1	pct.2	p_val_adj
1	Rpl41	1.36E-29	-1.143862273	1	1	4.22E-25
2	Rps29	2.08E-21	-0.880603537	0.977	0.946	6.46E-17
3	Rps27	9.47E-18	-0.874483462	1	0.973	2.94E-13
4	Rps28	3.40E-16	-1.018630657	0.909	0.973	1.06E-11
5	Rpl39	1.42E-11	-0.770234978	0.977	1	4.41E-07
6	mt-Nd3	3.25E-11	-1.095754423	0.841	0.919	1.01E-06
7	Rpl38	1.96E-10	-0.85883353	0.932	0.973	6.07E-06
8	Uba52	3.25E-10	0.524452126	0.795	0.73	1.01E-05
9	Rps21	7.92E-09	-0.729500546	0.977	0.973	0.000246
10	Rps3a1	1.15E-08	-0.059279833	1	0.973	0.000357
11	Rpl37a	7.04E-08	-0.692523285	0.955	0.973	0.002186
12	Rpl37	1.53E-07	-0.65346671	0.977	0.973	0.004738
13	Rplp2	8.23E-07	-0.689704088	0.955	1	0.025555
14	Pdcd4	1.69E-06	-1.018759626	0.477	0.865	0.052423
15	Atp1b3	2.01E-06	-1.179291223	0.591	0.865	0.062569
16	Tpt1	2.46E-06	-0.048868581	1	1	0.076447
17	Malat1	2.80E-06	-0.929975182	1	1	0.08691
18	Rpl19	5.17E-06	-0.12558959	0.977	1	0.160544
19	Metap2	7.00E-06	-0.83289621	0.636	0.892	0.217426
20	Gja1	1.27E-05	-1.516482496	0.523	0.865	0.39503
